# Next-Generation Molecular Diagnostics Development by CRISPR/Cas Tool: Rapid Detection and Surveillance of Viral Disease Outbreaks

**DOI:** 10.3389/fmolb.2020.582499

**Published:** 2020-12-23

**Authors:** Sonal Srivastava, Dilip J. Upadhyay, Ashish Srivastava

**Affiliations:** ^1^Amity Institute of Virology and Immunology, Amity University Uttar Pradesh, Noida, India; ^2^Amity University Uttar Pradesh, Noida, India

**Keywords:** Cas12, Cas13, isothermal amplification, Cas9, CRISPR associated proteins, NAT

## Abstract

Virus disease spreads effortlessly mechanically or through minute insect vectors that are extremely challenging to avoid. Emergence and reemergence of new viruses such as severe acute respiratory syndrome coronavirus 2 (SARS-CoV-2), H1N1 influenza virus, avian influenza virus, dengue virus, Citrus tristeza virus, and Tomato yellow leaf curl virus have paralyzed the economy of many countries. The cure for major viral diseases is not feasible; however, early detection and surveillance of the disease can obstruct their spread. Therefore, advances in the field of virus diagnosis and the development of new point-of-care testing kits become necessary globally. Clustered regularly interspaced short palindromic repeats (CRISPR)/CRISPR-associated protein (Cas) is an emerging technology for gene editing and diagnostics development. Several rapid nucleic acid diagnostic kits have been developed and validated using Cas9, Cas12, and Cas13 proteins. This review summarizes the CRISPR/Cas-based next-generation molecular diagnostic techniques and portability of devices for field-based utilization.

## Introduction

For the last two decades, the emergence and reemergence of new viral diseases have compromised healthcare and agriculture worldwide. They arise from new virus variants, which are generated by mutations or recombination, and show novel pathogenic and epidemiological properties. These viruses infect humans, animals, crops, and fishes and adversely affect economies. At the onset of the 21st century, three coronaviruses (CoVs) caused three major epidemics of respiratory distress syndrome, which comprise severe acute respiratory syndrome coronavirus (SARS-CoV) in 2003 in Guangdong, China; the Middle East respiratory syndrome coronavirus (MERS-CoV) in 2012 in Saudi Arabia; and the respiratory coronavirus, the novel coronavirus disease 2019 (COVID-19), which started from Wuhan province, China, and later extended its infection in South Korea, Japan, Italy, Iran, and USA and has further spread to India, Bangladesh, and Pakistan (Xu et al., 2004; Nassar et al., 2018; Abduljalil and Abduljalil, 2020). Ebola hemorrhagic fever, plague, Japanese encephalitis, avian influenza, yellow fever, and Zika virus disease are some of the major outbreaks in the history of human beings caused by viruses that came out at times and brought suffering to their lives (https://www.who.int/csr/don/archive/year/en/). Similarly, the Tobacco mosaic virus, Tomato spotted wilt virus, Tomato yellow leaf curl virus, Cucumber mosaic virus, Potato virus Y, African cassava mosaic virus, and Potato virus X are major plant pathogens that adversely affect economies each year (Scholthof et al., 2011). Some viral infections are difficult to treat and may require robust host defense mechanisms to suppress them. Therefore, the best way to manage viral diseases is early detection and prevention of their spread in the community.

For surveillance of emerging viruses, there is the requirement of reliable point-of-care testing (POCT) kits, which can be used at the site of infection. The recent outbreak of SARS-CoV-2 accelerates research in the field of diagnostics to develop rapid, accurate, and ultrasensitive kits. Nucleic acid-based testing (NAT) tools proved ultrasensitive and accurate diagnostics for multiplexed virus infection, thus major attempts are being performed to convert typical diagnostics into nucleic acid POCT tools. Popular NAT kits are based on quantitative real-time reverse-transcription polymerase chain reaction (qRT-PCR), which requires expensive infrastructure and trained manpower, therefore not feasible to utilize at the point of infection. Nowadays, recombinase polymerase amplification (RPA), loop-mediated isothermal amplification (LAMP), and clustered regularly interspaced short palindromic repeats (CRISPR)-based tools are popular among NAT tools. These tools may replace costly qRT-PCR-based testing systems. The CRISPR/CRISPR-associated protein (Cas) system is rapidly becoming well known for its powerful potential not only in therapeutics but also in getting a unique identity in the field of molecular diagnostics. Intense research efforts are being conducted around the globe to identify efficiently CRISPR's search function to be utilized in diagnostics. Recently, an improved understanding of other diverse CRISPR/Cas systems has expanded CRISPR's applications in disease diagnosis. The recent discovery of different classes of RNA-guided nuclease, Cas protein has unlocked the door to new tools that offer cost-effective and portable diagnostics through nucleic acid screening.

### Next-Generation Nucleic Acid-Based Testing Kit

The modern NAT kits are reliable and highly sensitive such as quantitative PCR kits for SARS-CoV-2 (Corman et al., 2020); however, they require expensive infrastructure and trained manpower. The qRT-PCR is an approach being used commonly for the diagnosis of viruses such as SARS-CoV-2 infection in upper and lower respiratory specimens at most diagnostic labs around the world (Corman et al., 2020). Due to the high cost of diagnosis per sample and unavailability at POCT facilities, these kits can be used at specified places and specimens need to be transported in protected cold chains to the point of action. Therefore, the availability of an affordable and portable diagnostic system may aid in large-scale screening of the affected population to halt the chain of viral infection. Other very popular virus diagnostic tools are LAMP and RPA, which show their potential in the diagnosis of major viruses in society. The design of these kits is portable with the requirement of very few or no instruments but requires multiplexing with separate technology for confirmation of the results.

LAMP, originally developed by Notomi et al. (2000), is a loop-mediated isothermal nucleic acid amplification approach that has been evaluated for the detection of several viruses including SARS-CoV-2 (Huang et al., 2020; Park et al., 2020), influenza virus (Ahn et al., 2019), Newcastle disease virus (Pham et al., 2005), foot-and-mouth disease virus (Farooq et al., 2015), chikungunya virus (Lopez-Jimena et al., 2018), plum pox virus (Hadersdorfer et al., 2011), potato leafroll virus (Ahmadi et al., 2013), Citrus tristeza virus (Selvaraj et al., 2019), Potato virus X (Jeong et al., 2015), tomato torrado virus (Budziszewska et al., 2015), and Rice black-streaked dwarf virus (Du et al., 2019). LAMP is a highly sensitive method for the detection of several viruses; however, it initially required careful primer designing and standardization. Similarly, RPA, an isothermal alternative of polymerase chain reaction (PCR), is a sensitive molecular diagnostic technology, which is rapid, portable, and can be multiplexed for several pathogens (Rostron et al., 2019). RPA has been used as reliable and rapid diagnostics for several viruses such as the human immunodeficiency virus (Crannell et al., 2014), Zika virus (Vasileva Wand et al., 2019), avian influenza A (Waheda et al., 2015), rabies virus (Coertse et al., 2019), Potato virus Y and Wheat dwarf virus (Glais and Jacquot, 2015), Tomato yellow leaf curl virus (Londoño et al., 2016), Apple stem grooving virus (Kim et al., 2018), and Banana bunchy top virus (Kapoor et al., 2017). Although these techniques proved better POCT techniques, detailed analysis of these molecular diagnostic mechanisms shows their low sensitivity and low throughput (Huang et al., 2020). Therefore, CRISPR/Cas-based diagnostics may take lead to become ultrasensitive diagnostic tools. Here we are summarizing the role of CRISPR/Cas technology in the development of rapid and ultrasensitive convergent and portable devices (Supplementary Figure 1).

## Application of CRISPR/Cas In Molecular Diagnostics

CRISPR sequence together with Cas nucleases forms the basis of a technology known as CRISPR/Cas that is used for genome editing. CRISPR is a family of DNA sequences found within the genome of prokaryotic organisms. Cas is the most commonly used endonuclease in gene-editing tools and utilizes a guide RNA to bind to a complementary DNA sequence, which is subsequently cleaved through Cas endonuclease activity. Protospacer adjacent motif (PAM) is a 2–6-base pair DNA sequence immediately following the DNA sequence targeted by the Cas nuclease in the CRISPR bacterial adaptive immune system. The most commonly studied endonuclease belongs to the type II CRISPR system, i.e., Cas9 that targets phage's DNA, while the type V CRISPR system, i.e., Cas12 also targets DNA, and type III and type VI groups exhibit RNA targeting activity, which includes Cas13 (Ford, 2019).

### Role of CRISPR-Associated Proteins and Single-Guide RNA in Target Detection

Cas9 is a unique enzyme that gets recruited by a trans-activating CRISPR RNA (tracrRNA) and guided by single-guide RNA (sgRNA), which facilitates its specific binding to target double-stranded DNA (dsDNA). Binding of sgRNA activates nuclease activity of Cas9, which makes a blunt double-stranded DNA break *in vitro* (Mehravar et al., 2019). The presence of PAM site on the target DNA helps in Cas9 binding. The most commonly used *Streptococcus pyogenes* Cas9 (SpCas9) recognizes 5' NGG 3' as PAM, while *Francisella novicida* Cas9 (FnCas9) recognizes 5' NGG 3' as PAM sequence on target site (Wiedenheft et al., 2012). Another form of Cas9 protein, that is, the dCas9 (nuclease-deficient Cas9) is being utilized to edit gene expression when being applied to the transcription binding site of the desired section of a gene. This special feature of Cas9 can also be utilized for the diagnosis of nucleic acids (Zhou W. et al., 2018). Another class of Cas protein (Class 2 type V) is Cas12 (Cpf1), which is a proficient enzyme that creates staggered cuts in dsDNA. Cas12 requires a CRISPR RNA (crRNA) that leads to an increase in multiplexing ability and recognizes a T-rich PAM instead of a G-rich PAM and generate dsDNA breaks with staggered 5′ ends. CRISPR-Cas12a possesses the cis-trans cleavage activity of ssDNA, which is being used these days for the diagnosis of various pathogens (Chen et al., 2018).

A new class of Cas, Cas13 (C2c2) nuclease, is a unique protein that has specific recognition and cleavage activity for complementary RNA and known for trans cleavage or collateral cleavage of nearby RNA (Abudayyeh et al., 2016). This feature played an important role in the diagnosis of viruses and other mRNAs *in vitro* (Kellner et al., 2020). The class of protein contains Cas13a, Cas13b, Cas13c, and Cas13d, which can be engineered to cleave target RNA transcripts (O'Connell, 2019). Once it gets activated by ssRNA sequence bearing complementarity to its crRNA spacer, it unleashes a nonspecific RNase activity and destroys nearby RNA regardless of its sequences. This property has been harnessed *in vitro* for precision diagnostics. Here, we are highlighting major CRISPR/Cas-based assays developed for diagnostic purposes.

#### Cas9-Based Diagnostics for Viruses

Application of Cas9 in diagnostics development has been exploited extensively, as well as its ability to create single-strand DNA nicks, truncation of DNA, Cas9-induced amplification, or specific binding of dCas9 to target DNA. The Zika virus outbreak led Pardee et al. (2016) to first use Cas9 as a diagnostic tool for viruses, where they used nucleic acid sequence-based amplification (NASBA), an isothermal amplification technique based on CRISPR cleavage (NASBACC). Here, if sgRNA/Cas9 finds a target site along with the PAM sequence, it creates nicks in DNA that produce truncated RNA through *in vitro* transcription. While the non-targeted one produces complete RNA, which activates Toehold reaction. Therefore, this technique can be utilized potentially to distinguish close viral strains if sgRNA would be designed based on unique PAM sites. This technique with the application of Cas9 can uniquely distinguish closely related Zika virus strains *in vitro*, and the detection of the target RNA was indicated by a color change in the paper disc from yellow to purple (Pardee et al., 2016). A unique method, CRISPR/Cas9-triggered nicking endonuclease-mediated strand displacement amplification (CRISDA), was also introduced for ultrasensitive detection of dsDNA in addition to fluorescence technique (Zhou W. et al., 2018). To do so, it utilizes Cas9 proteins for recognition of a highly specific target and creates unique nicks in non-target DNA, which is further amplified by DNA polymerases *in vitro*. A unique site-specific NAT kit, CRISPR/Cas9-triggered isothermal exponential amplification reaction (CAS-EXPAR), was also developed, which utilizes the target-specific nicking activity of Cas9 and nicking endonuclease (NEase). Unlike CRISDA, this technique does not require any external primers and hence proved more specific for the mutated targets (Huang et al., 2018).

Further, CRISPR/Cas9-mediated lateral flow nucleic acid assay (CASLFA) was developed for the diagnosis of African swine fever virus (ASFV). In this diagnostic kit, CRISPR/Cas9-based ultrasensitive lateral flow nucleic acid diagnosis of ASFV was demonstrated (Wang et al., 2020). The establishment of inactive dead Cas9 (dCas9), which selectively binds to target DNA without nick/cleavage, enhances the popularity of this protein in the development of CRISPR/Cas9-based diagnostic kits. Azhar et al. (2020) have developed a dCas9-based COVID 19 diagnostic kit, which uses FnCas9 and named FNCAS9 Editor-Linked Uniform Detection Assay (FELUDA). Here, they used fluorescein amidites (FaM) tracrRNA-sgRNA and anti-FAM antibody conjugate with nanoparticles and demonstrated it as a POCT kit for versatile applications including rapid diagnosis during infectious disease outbreaks like COVID-19. Combined with RT-RPA amplification and specific binding of dFaCas9, they developed a lateral flow device for COVID-19 virus diagnosis (Table 1). Further application of these kits can be extended for other emerging human, animal, or plant viruses.

**Table 1:** Various CRISPR-associated protein-based diagnostic platforms for virus diagnosis and their mode of detection.

**Nuclease**	**Platform**	**Primary amplification method**	**Detection method**	**Mechanism**	**References**
Cas9	NASBACC	NASBA	Electronic optical reader	PAM recognition and cleavage to activate Toehead switch	(Pardee et al., 2016)
	CRISDA	PCR	Fluorescence spectrophotometer	Cas9 creates nicks at the border and the target gene amplifies through external primers	(Zhou W. et al., 2018)
	CAS-EXPAR	EXPAR	Real-time fluoresces monitoring	Cas9 creates nicks and NEase produces ssDNA internal primers cyclically	(Huang et al., 2018)
	CASLFA	PCR	Paper-based LF device	Cas9/sgRNA complexes with target DNA and detected through AuNP-DNA probes	(Wang et al., 2020)
	FELUDA	RPA/PCR	Paper-based LF device	gRNA-dFnCas9 RNP complexes with target DNA and detected colorimetric LF device	(Azhar et al., 2020)
Cas12	DETECTR	LAMP	Paper-based LF device	Cas12a-crRNA complex binds and cleaves a dsDNA and detects fluorescence in collateral cleaved probe DNA	(Broughton et al., 2020)
	HOLMES	PCR	Fluorescence spectrophotometer	Cas12a-crRNA complex binds and cleaves a dsDNA and detects fluorescence in collateral cleaved probe DNA	(Li et al., 2018)
	HOLMESv2	LAMP	Fluorescence spectrophotometer	HOLMES upgraded; LAMP and Cas12b trans-cleavage integrated into one step	(Li et al., 2019)
	AIOD-CRISPR	PCR	LED blue light illuminator	One-pot collateral cleavage reaction system and colorimetric detection	(Ding et al., 2020)
	iSCAN	LAMP	Fluorescence visualization in UV light and LF device	CRISPR-Cas12a-based collateral cleavage and fluorescence-based detection	(Ali et al., 2020)
Cas13	SHERLOCK	RPA	Fluorescence spectrophotometer/ Paper based LF device	crRNA/Cas13 targets an ssRNA and cleaved fluorescent ssRNA probe collaterally	(Gootenberg et al., 2017; Kellner et al., 2020)
	SHERLOCKv2	RPA	LF device	Upgraded SHERLOCK; High quantification and sensitivity	(Gootenberg et al., 2018)
	CREST	RPA/PCR	Fluorescence spectrophotometer/Paper based LF device		(Rauch et al., 2020)
	CARMEN	PCR	Fluorescence spectrophotometer	SHERLOCK method in one array enables more than 4,500 nucleic acids.	(Ackerman et al., 2020)
	CARVER	PCR	Fluorescence spectrophotometer	Most advanced system to diagnose a sample using the SHERLOCK method, treat a viral infection, and measure the effectiveness of the treatment	(Freije et al., 2019)

*CARMEN, Combinatorial Arrayed Reactions for Multiplexed Evaluation of Nucleic acids; CARVER, Cas13-assisted restriction of viral expression and readout; CREST, Cas13-based, Rugged, Equitable, Scalable Testing; CRISDA, CRISPR/Cas9-triggered nicking endonuclease-mediated strand displacement amplification; CRISPR, clustered regularly interspaced short palindromic repeats; DETECTR, DNA Endonuclease Targeted CRISPR Trans Reporter; FELUDA, FNCAS9 Editor-Linked Uniform Detection Assay; HOLMES, one-HOur Low-cost Multipurpose highly Efficient System; LAMP, loop-mediated isothermal amplification; PAM, protospacer adjacent motif; RNP, ribonucleoprotein; RPA, recombinase polymerase amplification; SHERLOCK, specific high-sensitivity enzymatic reporter unlocking*.

#### Cas12-Based Diagnostics

After the success story of Cas9 in gene editing and diagnostics development, extensive researches were performed around the world for the discovery of new Cas effectors. Cas12 and Cas13 are the most studied effector proteins after Cas9, which have great application in ssRNA and ssDNA cleavage, therefore, they have wide application in diagnostics development (Chen et al., 2018). Cas12 is a type V CRISPR-associated effector protein, which is being used for CRISPR-based diagnosis. Cas12a effector (Cpf1) requires smaller crRNA (CRISPR RNA) without tracrRNA and processes its crRNA with its RNase activity and cleaves DNA by RuvC domain. Among Cas12a–g, Cas12g (~800 kb) effectors are the smallest, and once activated, they can cleave ssDNA and RNA in trans; therefore, they are most suitable for diagnosis (Rusk, 2019). CRISPR/Cas12-based diagnostic kits were also made in lateral flow devices as well as fluorimeter-based diagnosis. Cas12a is the most explored effector for diagnostics. The Cas12a-based system utilizes ssDNA probes in place of RNA probes, therefore, it is more stable in field conditions. Unlike Cas13-based detection, this technique does not require synthesis of RNA from amplified target DNA through *in vitro* transcription, which reduces the cost, technical errors, and time.

These diagnostic kits are based on specific binding of Cas12 protein with target DNA and collateral cleavage activity of ssDNA. Commonly, the isothermal amplification of the target virus gene is performed, added with virus-specific crRNAs, Cas12 effectors, and ssDNA probes and incubated in the tube. If the Cas12 binds with target dsDNA, it gets activated and cleaves the amplified DNA as well as probes that reflect the fluorescence. This fluorescence can be measured by fluorometer or coupled with paper-based detection systems. Coupled with isothermal amplification, one-HOur Low-cost Multipurpose highly Efficient System (HOLMES) and DNA Endonuclease Targeted CRISPR Trans Reporter (DETECTR) are two major CRISPR/Cas12-based diagnostic systems, which have been applied worldwide (Li et al., 2018). For standardization of the HOLMES-based diagnostic assay, around 10 different bacterial Cas12a were evaluated (Supplementary Table 1), and among them, LbCas12a, OsCas12a, Lb5Cas12a, and FnCas12a were found suitable for diagnosis of DNA viruses such as Pseudorabies virus and RNA virus such as the Japanese encephalitis virus (Li et al., 2018). With the extension of the HOLMES, the more advanced HOLMESv2 was developed, which utilizes the action of Cas12b. In HOLMESv2, Cas12b was standardized for various applications such as single-nucleotide polymorphism (SNP) detection and simplifies virus RNA amplification and detection and quantification of DNA methylation with bisulfite treatment (Li et al., 2019). RNA-guided Cas12a was also used in DETECTR, which specifically identified the human papillomavirus (HPV) in the specimen (Chen et al., 2018). A recent report by Broughton et al. (2020) demonstrated CRISPR/Cas12-based detection of SARS-CoV-2 utilizing DETECTR assay and claimed as a visual and faster alternative to the SARS-CoV-2 real-time RT-PCR assay. In the current COVID-19 situation, the new Cas12a-based rapid and highly sensitive kits, All-in-One Dual CRISPR-Cas12a (AIOD-CRISPR) and iSCAN (*in vitro* Specific CRISPR-based Assay for Nucleic acids detection), are also introduced, which successfully diagnose both SARS-CoV-2 and HIV (Ali et al., 2020; Ding et al., 2020). These techniques are also based on the collateral cleavage activity of ssDNA probes. The potential role of this protein was also reported in the diagnosis of white spot syndrome virus in shrimps (Chaijarasphong et al., 2019). Cas12-based diagnostics play an important role in the direct diagnosis of DNA viruses without undergoing RNA transcription, which makes it popular for the diagnosis of both DNA and RNA viruses (Table 1).

#### Cas13-Based Diagnostics

Cas13 is a unique RNA-guided protein of class 2 type VI that possesses RNA activity, and once activated with binding to target RNA, cleaves nearby RNAs in trans (Abudayyeh et al., 2016). Similar to HOLMES, Cas13-based detection systems require isothermal amplification of the target genome, crRNAs, and fluorescent ssRNA probes. One additional step for the synthesis of *in vitro* RNA is required for this technique. Once crRNA binds with amplified RNA, Cas13 cleaves it and starts collateral cleavage of nearby ssRNA probes. The fluorescence can be measured and quantified by various techniques. CRISPR/Cas13 has played a vital role in viral diagnostics using platform specific high-sensitivity enzymatic reporter unlocking (SHERLOCK) (Gootenberg et al., 2017). The target RNA either detected directly in one step or coupled with RT-RPA isothermal amplification in two steps. Cas13 binds with the target RNA sequences, cleaves surrounding RNA transcripts including the RNA reporter due to its nonspecific cleavage property, which results in the emission of a fluorescent signal that is recorded by the detector (Kellner et al., 2020). The SHERLOCK system has been adopted for the diagnosis of several viruses such as dengue virus, Zika virus, SARS-CoV-2 of human, and White spot syndrome virus of shrimps (Sullivan et al., 2019; Kellner et al., 2020). For diagnosis of SARS-CoV-2, CRISPR/Cas13-based diagnostics have explored much for nucleic acid-based diagnosis. Researchers have developed a protocol for the application of SHERLOCK for COVID-19 diagnosis (Zhang et al., 2020). A novel method based on Cas13-based effector that is Rugged, Equitable, Scalable Testing (CREST) has also been introduced for SARS-CoV-2 diagnosis, which has low-cost instrumentation without sacrificing detection sensitivity (Rauch et al., 2020). A highly multiplexed method for pathogen detection was developed by a combination of Combinatorial Arrayed Reactions for Multiplexed Evaluation of Nucleic acids (CARMEN) with Cas13 and demonstrated to detect 169 human viruses including SARS-CoV-2 (Ackerman et al., 2020). Freije et al. (2019) introduced a Cas13-assisted restriction of viral expression and readout (CARVER), which can detect lymphocytic choriomeningitis virus (LCMV), influenza A, and other RNA viruses (Table 1). Cas13-based diagnostics seems promising for future nucleic acid-based diagnostics due to its role in multiplexing and throughput diagnostic technique development.

## Convergence and Portability of Nucleic Acid-Based Testing Kits

CRISPR/Cas-based kits were initially developed based on their cleavage or specific binding activities with target nucleic acids. Cas12- and Cas13-based kits work on the principle of collateral cleavage of ssDNA and ssRNA, respectively, while Cas9-based kits work on cleavage or binding with the nucleic acid. To enhance sensitivity, such kits need to be multiplexed with nucleic acid amplification techniques such as PCR, RPA, LAMP, NASBA, etc. The addition of such techniques adds new steps in diagnosis, which also compounds the time of diagnosis and technical errors. Another concern arises with the availability of resources and infrastructure for field-based diagnoses such as qRT-PCR, fluorescence detector, or transilluminator. As we discussed above, researchers are developing new techniques that are focusing on the multiplexing of the isothermal amplification and CRISPR/Cas-based diagnosis steps and successfully demonstrated the detection of single nucleotide change. Further, several developments were made to make portable kits suitable for POC testing, which is summarized below.

### Paper-Based Lateral Flow Assay Kit

**Figure 1:**
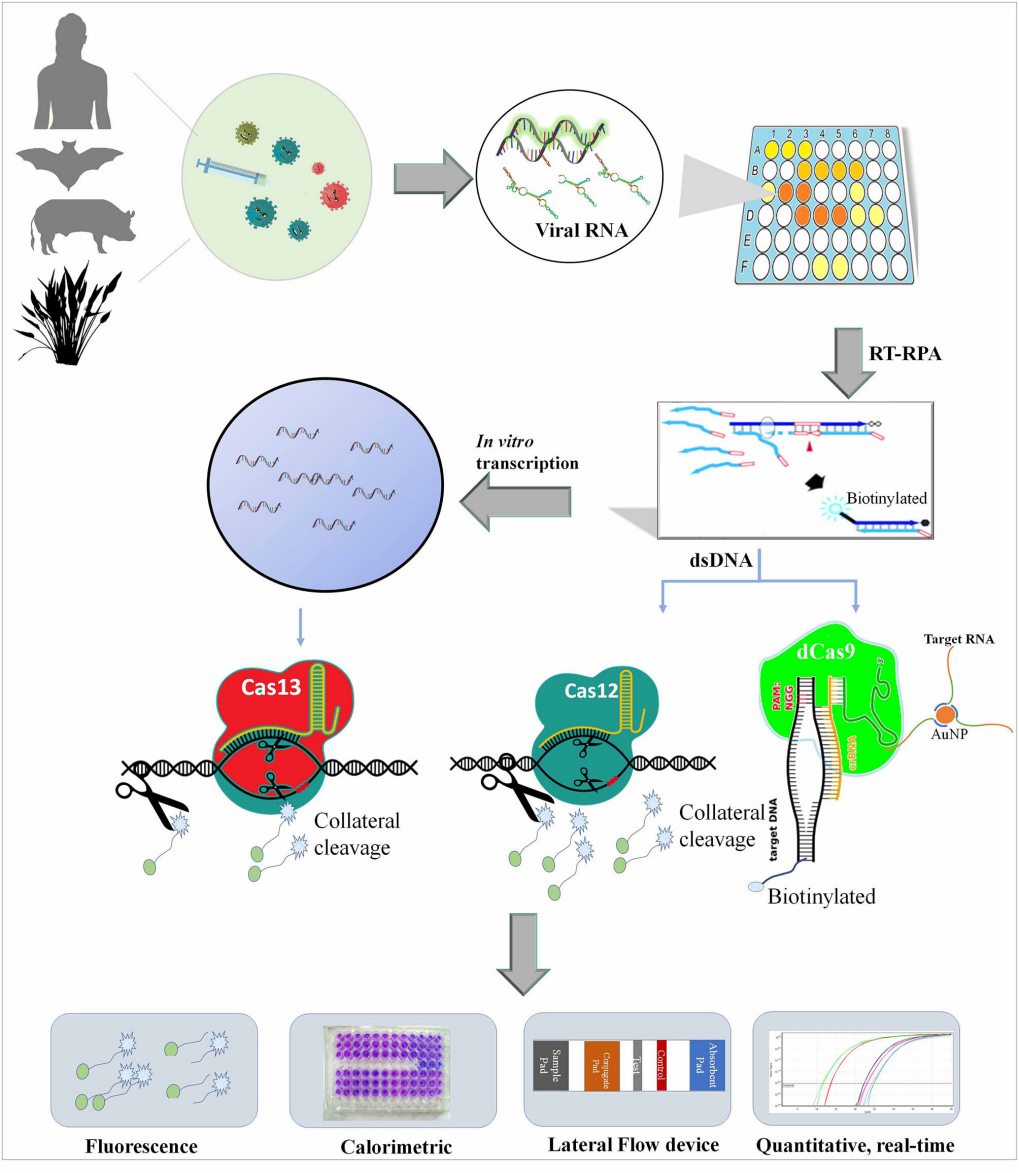
Shows the diagnosis of viruses from various sources using Cas9, Cas12, and Cas13 and multiplexing with other nucleic acid-based testing (NAT) kits.

Lateral flow assay (LFA) kits are the most popular POCT kits, which are generally based on the molecular affinity of various molecules such as antigen–antibodies, streptavidin-biotin, etc. It is the simplest device for POCT for various pathogens, diseases, or hormonal changes in humans, animals, or plants. These colorimetric devices are developed on paper material such as nitrocellulose paper and generally contain one or two test lines and a control line and do not require any specialized electronic device to visualize the results. CRISPR/Cas9-based CASLFA (Wang et al., 2020) and FELUDA (Azhar et al., 2020) techniques are assembled to LFA kits with the use of gold nanoparticles (AuNPs) fused with antibodies or other interacting molecules. In CASLFA, the RPA/PCR is performed using biotinylated primers and the amplified target gene is mixed with sgRNA, Cas9, and AuNP probe and loaded onto the sample pad of the LFA device. Streptavidin-coated test lines and complementary DNA probe hybridized control pad specifically bind with biotinylated sample mix and AuNP-DNA probes and show color due to the accumulation of AuNP at test line and control pad. Based on color development at the test line and control pad, a target virus can be detected (Wang et al., 2020). Similarly, in FELUDA, chimeric guide RNA was mixed in a tube with 3'-FAM-labeled ribonucleoprotein (RNP) complex and FAM antibody-linked gold nanoparticles and loaded to a paper strip (Azhar et al., 2020). Tsou et al. (2019) have demonstrated HPV DNA diagnosis by CRISPR/Cas12a-based LFA kit. Here, they used the trans-cleavage activity of Cas12a to cleave the FAM-fluorescent biotinylated ssDNA probe, which resulted in the binding of biotin to streptavidin without FAM fluorescence at the control line. This allows anti-FAM-AuNP to flow freely from the control line and bind with anti-rabbit antibody immobilized at the test line and show a pink color deposition, which is an indicator of positive infection (Kanitchindaa et al., 2020). A similar strategy was applied for LFA device development for Cas13a-based diagnosis of viral nucleic acid in the famous SHERLOCK technique (Kellner et al., 2020). LFA strips may play a great role in the surveillance of disease outbreaks such as COVID-19 (Figure 1).

### Paper-Based Microfluidic Devices

Paper-based microfluidic devices (μPAD) are an advanced form of LFA, emerged as cost-effective and portable platforms for POCT, and are primarily made up of fibers and direct fluid from an inlet to the outlet through imbibition and could be run by applying a voltage into a triangular sheet of wet paper. English et al. (2019) have presented paper-based polyacrylamide-DNA technologies that show promise as POCT for the Ebola virus, as they are robust, inexpensive, equipment-free, and rapid. The Cas12a cleaves trans ssDNA, which is loaded onto gel to convert biological information to material and can be used in a polyacrylamide-DNA hydrogel operating as a fluidic valve with an electrical readout for remote signaling (English et al., 2019). The diagnosis of microRNAs was made easy by Cas13-based integrated microfluidic electrochemical biosensor. In this approach, no multiplexing of nucleic acid amplification method was required, which makes this technique inexpensive, rapid, and target amplification-free tool for nucleic acid-based diagnostics, therefore, proving significant for the diagnosis of SARS-CoV-2 (Ramachandra et al., 2020).

### Fluorescence-Based Detection

The setup for paper-based LFA and microfluidic devices are very tricky to perform in the laboratory and required good experimental skills. Therefore, an easy and POCT alternative for this kit is the fluorescence-based diagnosis of CRISPR/Cas-cleaved fluorescent molecules. These days, many battery-operated and inexpensive fluorescence detectors are available, which can be used in non-laboratory conditions. Such devices can be developed by the use of LED and filter foils, which can detect up to a lower limit of detection of approximately 6.8 nM fluorescein (Katzmeier et al., 2019). In the Cas9-based diagnosis method, CRISDA, the dsDNA is nicked at two positions by Cas9 and exposed non-target DNA strands primed with a pair of probe primers. The amplified products were fused with streptavidin and Cy5-labeled probes, which were further isolated through magnetic pull-down method and pathogen detected by a fluorometer (Zhou L. et al., 2018). The Cas12- and Cas13-based methods work differently by detecting cleaved non-target probes in collateral cleavage once bound with target nucleic acid. SHERLOCK (Kellner et al., 2020), DETECTR (Chen et al., 2018, Broughton et al., 2020), HOLMES and HOLMESv2 (Li et al., 2018, 2019), and many other techniques commonly use fluorescent-based assays to develop POC diagnostic kits for viruses, and such methods are frequently being utilized for SARS-CoV-2 diagnosis (Guo et al., 2020) (Figure 1).

### Toehold Switch RNA Sensors

Toehold switches are synthetic RNAs that act as mRNAs whose role is to switch between the start and stop of the translation. In a normal situation, a toehold forms a hairpin loop structure that blocks the translation of the reporter gene by checking the ribosome movement. Pardee et al. (2016) first utilize this switch for Cas9-based diagnosis of two different strains of the Zika virus where PAM sequences play an important role in the recognition of single nucleotide change. In this technique, the binding of a trans-acting trigger RNA synthesized due to translation of non-truncated viral DNA activates toehold switch and detected through the colorimetric device, while Cas9-cleaved truncated RNA could not activate it. Further progress of this technique in multiplexing with CRISPR/Cas9-based diagnosis is being evaluated and recognized as a powerful tool for disease diagnosis (Qiu et al., 2018).

## Future Perspectives and Conclusions

The recent development in the field of molecular diagnostics presented the positive side of the COVID-19 outbreak. This incidence suggests that the only way to treat a virus disease is its early diagnosis in the mass population and containment of the pathogen. CRISPR/Cas-based diagnostics may prove to be a better option to develop ultra-sensitive, inexpensive, and rapid non-laboratory-based detection kits for several viruses and other pathogens. In this review, we have summarized the several kits such as CASLFA, FELUDA, DETECTR, HOLMES, SHERLOCK, etc., which are developed by the CRISPR-associated nucleases Cas9, Cas12, or Cas13. We also focus on techniques used for the portability of the devices to deploy them into field conditions.

## Author Contributions

AS has developed the concept. SS and AS has written manuscript. DU evaluated and corrected the text. All authors contributed to the article and approved the submitted version.

## Conflict of Interest

The authors declare that the research was conducted in the absence of any commercial or financial relationships that could be construed as a potential conflict of interest.

## Supplementary Material

Supplementary Figure 1 Schematic representation of nucleic acid-based testing (NAT) methods, recombinase polymerase amplification (RT-RPA), and loop-mediated isothermal amplification (RT-LAMP) to diagnose RNA viruses *in vitro*.
