# Supplementary material for: Next-Generation Molecular Diagnostics Development by CRISPR/Cas Tool: Rapid Detection and Surveillance of Viral Disease Outbreaks
Source: Front Mol Biosci. 2020 Dec 23;7:582499. doi: 10.3389/fmolb.2020.582499 (PMC7785713; doi:10.3389/fmolb.2020.582499)
Supplement: Supplementary file 1 [file Table_1.DOCX]

**Table S1:** List of bacterial strains standardized for HOLMES (one-HOur Low-cost Multipurpose highly Efficient System) based diagnostic assay (Li et al ., 2018).

| **S. No.** | **Bacterial Strain** | **Cas12a** |
| --- | --- | --- |
| 1. | *Francisella tularensis* | FnCas12a |
| 2. | *Acidaminococcus* sp. | AsCas12a |
| 3. | *Lachnospiraceae* bacterium | LbCas12a, Lb5Cas12 |
| 4. | *Oribacterium* sp | OsCas12a |
| 5. | *Helcococcus kunzii* | HkCas12a |
| 6. | *Thiomicrospira* sp | TsCas12a |
| 7. | *Bacteroidales* sp. | BbCas12a, BoCas12a |
| 8 | *Lachnospiraceae* bacterium | LbCas12a |
