# Supplementary figures and images for: Next-Generation Molecular Diagnostics Development by CRISPR/Cas Tool: Rapid Detection and Surveillance of Viral Disease Outbreaks
Source: Front Mol Biosci. 2020 Dec 23;7:582499. doi: 10.3389/fmolb.2020.582499 (PMC7785713; doi:10.3389/fmolb.2020.582499)

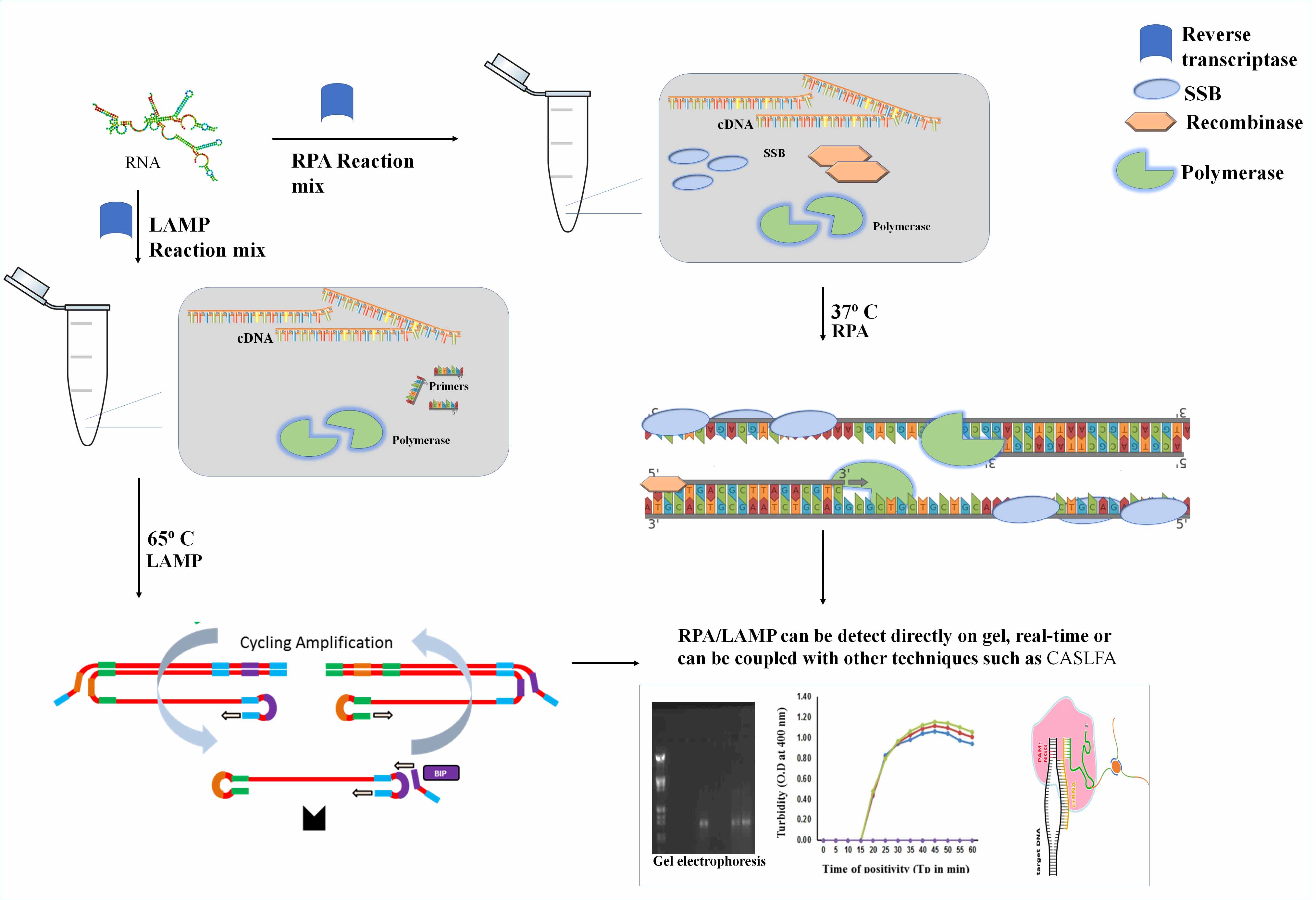

Supplement: Supplementary Figure 1 — Schematic representation of nucleic acid-based testing (NAT) methods, recombinase polymerase amplification (RT-RPA), and loop-mediated isothermal amplification (RT-LAMP) to diagnose RNA viruses in vitro. [file Image_1.JPEG]
